# Increased Levels of miRNA-146a in Serum and Histologic Samples of Patients with Uveal Melanoma

**DOI:** 10.3389/fphar.2016.00424

**Published:** 2016-11-15

**Authors:** Andrea Russo, Rosario Caltabiano, Antonio Longo, Teresio Avitabile, Livio M. Franco, Vincenza Bonfiglio, Lidia Puzzo, Michele Reibaldi

**Affiliations:** ^1^Department of Ophthalmology, University of CataniaCatania, Italy; ^2^Unità di Anatomia Patologica, Department Gian Filippo Ingrassia, University of CataniaCatania, Italy

**Keywords:** microRNAs, serum, enucleation, uveal melanoma, FFPE

## Abstract

**Purpose:** To analyze MiRs expression in serum of UM patients, respect to healthy donors, and to compare this data with MiRs expressed in formalin-fixed, paraffin-embedded UM samples.

**Methods:** Expression profile of 754 miRNAs was performed in serum of patients with uveal melanoma who underwent primary enucleation. The level of miRNAs increased in serum was individually analyzed on FFPE UM samples and compared to choroidal melanocytes from unaffected eyes.

**Results:** Fourteen patients with uveal melanoma were included in the study. We found 8 serum miRNAs differentially expressed compared to normal controls: 2 upregulated miRNAs (miRNA-146a, miR-523); 6 downregulated miRNAs (miR-19a, miR-30d, miR-127, miR-451, miR-518f, miR-1274B). When data on upregulated miRNAs were singularly validated only a significant overexpression of miRNA-146a was found. A statistically significant upregulation of miRNA-146a was also found on FFPE UM samples, compared to choroidal melanocytes from unaffected eyes.

**Conclusions:** miRNA-146a is increased in serum of patients with UM and in FFPE tumor samples. Further studies will show if it could be considered a potential marker of UM in the blood.

## Introduction

Uveal melanoma (UM) is the most common primary intraocular malignancy in adults with an incidence in the United States of 5.1 cases per million (Singh et al., 2011). It has been estimated that even with appropriate treatment and close follow-up, 40–50% of patients with UM die from metastatic disease; the liver is involved in up to 90% of individuals and the median survival is 4–5 months (Kujala et al., 2003). Considering that an early detection could improve the survival rates of UM, a precise diagnostic test for early melanoma detection could be therefore useful. The underlying molecular biology of UM is complex and involves interactions between networks of genes, signaling pathways, and gene regulatory mechanisms, and a better understanding of these underlying molecular mechanisms is essential for translational research. Most UM cases are correctly diagnosed through ophthalmoscopy, ultrasonography, fundus fluorescein angiography, indocyanine green angiography, and magnetic resonance imaging (Russo et al., 2015). However, clinical presentation, size of lesion, opacity of refractive media, intraocular hemorrhage, and other factors may cause false negative or positive diagnosis.

Effective molecular diagnostic markers for this tumor have not been described. MicroRNAs (miRNAs) are a class of non-coding small RNAs that regulate gene expression by targeting specific mRNAs for degradation and/or translational repression and are involved in a large number of physiological and pathological processes. A growing body of research has demonstrated the important role of miRNAs in the initiation, progression, and metastasis of cancer including melanoma (Lynam-Lennon et al., 2009; Babashah and Soleimani, 2011; Babashah et al., 2012; Babashah, 2014). They influence cancer development by regulating transcription and translation of tumor suppressor genes and oncogenes. Several miRNAs were discovered in various body fluids including the serum, plasma, saliva, tears, breast milk, and urine. Various methods (e.g., qRT-PCR, northern blot, microarray, and deep sequencing) can be used to detect these extracellular and secretory miRNAs.

Circulating miRNAs fulfill a number of criteria as ideal biomarkers: accessibility through non-invasive methods, high degree of specificity and sensitivity, ability to differentiate pathologies, long half-life within samples, rapid, and accurate detection (Chen et al., 2008; Gilad et al., 2008; Mitchell et al., 2008). Our group previously showed that the expression of circulating miRNAs in Vitreous Humor (VH) is altered in different eye pathologies, including UM and in particular that miRNA-146a was overexpressed more than three-fold in VH from patients with uveal melanomas (Ragusa et al., 2013, 2015).

Unfortunately, detection of circulating miRNAs in VH for diagnostic purposes is not currently feasible because vitreous sampling (i.e., vitrectomy) is an unpleasant and invasive method.

The aim of this work was to analyze MiRs expression in serum of UM patients, respect to healthy donors, and to compare this data with MiRs expressed in formalin-fixed, paraffin-embedded UM samples. This data might suggest the possibility to screen the blood of uveal melanoma patients and consider a specific miRNA (146a) as a potential blood marker of UM.

## Materials and methods

Patients were included in the study prospectively and consecutively if they underwent primary enucleation for choroidal melanoma, at the University of Catania, Italy between October 2012 and April 2014.

This study was conducted in accordance with recommendations of the local ethic committee (“Comitato Etico Catania1”) and the Helsinki Declaration. Before the procedures written informed consent was obtained from all research participants in the study.

All patients underwent thorough ophthalmologic evaluations of the choroidal lesions, including indirect ophthalmoscopy, standardized A-scan and B-scan echography, fundus fluorescein angiography, and indocyanine green angiography. Multidetector computed tomography (MDCT) examination of chest, abdomen and pelvis was performed in order to assess that no metastatic disease was present.

Following the diagnosis of UM, eligible patients for enucleation were selected based on larger basal tumor diameter and height; as other methods were considered unlikely to conserve the eye and useful vision without causing excessive morbidity (Damato and Lecuona, 2004). The serum samples were collected before the enucleation procedure, which was performed at the Eye Clinic of the University of Catania.

Parameters considered were: gender, age at diagnosis, location of the tumor, thickness, largest diameter, cell type, extrascleral extension, occurrence of metastasis and pathological TNM stage (Table 1).

**Table 1:** **Demographics, tumor parameters, pathological stage in UM patients**.

**Sex**	**Age**	**Location**	**Thickness (mm)**	**Largest diameter (mm)**	**Cell type**	**Extrascleral extension**	**Metastasis**	**Pathological T stage**
F	49	Choroid/cil.body	15.93	19.7	Mixed	N	N	pT4b
M	67	Choroid	16.27	20.8	Spindle	N	N	pT4b
F	54	Choroid/cil.body	11.8	20.1	Mixed	N	N	pT4b
F	67	Choroid/cil.body	15	20	Spindle	N	N	pT4b
F	67	Choroid	14.5	16	Spindle	N	N	pT4a
F	72	Choroid/cil.body	15.5	19.2	Mixed	N	N	pT4b
F	67	Choroid	11	17.5	Mixed	N	N	pT3b
F	59	Choroid	16.9	17	Mixed	N	N	pT4a
M	52	Choroid	15.5	19.3	Spindle	N	N	pT4b
F	29	Choroid	16.4	19.2	Mixed	N	N	pT4a
F	83	Choroid/cil.body	17.8	21.4	Mixed	N	N	pT4a
M	48	Choroid	14.8	19.6	Spindle	N	N	pT4a
F	69	Choroid/cil.body	16.7	20.3	Spindle	N	N	pT4b
M	72	Choroid	14.2	17.6	Mixed	N	N	pT4a

### Serum sampling

All enrolled patients underwent fasting venous blood sampling. We analyzed serum from 14 healthy donors as controls. Blood samples were obtained by vein puncture using dry vacutainer tubes (BD Biosciences, Italy). The samples were processed for serum isolation within 2 h after withdrawal. Whole blood was left to stand for 30′ at 20°C before being centrifuged at 3000 rpm for 15′ at 4°C. Serum was divided into aliquots, and stored at −80°C until analysis.

### Tumor samples and clinico-pathological data

Histological examination was performed at the Unit of Anatomical Pathology, Department G.F. Ingrassia, University of Catania, Catania, Italy. Cases of iris melanoma, with incomplete patient records or without representative tumor tissue in paraffin blocks, were excluded. Fourteen patients who underwent primary enucleation were selected for miRNA analysis (Table 1). Controls for mRNAs in the ocular tissue were choroidal melanocytes from five unaffected eyes.

### RNA isolation from serum and miRNAs profiling by TaqMan low density array

Serum samples were centrifuged at 2000 rpm for 10′ to pellet any circulating cell or debris. miRNAs were extracted from 400 μl of serum samples by using Qiagen miRNeasy mini kit (Qiagen, GmbH, Hilden, Germany), according to Qiagen supplementary protocol for purification of small RNAs from serum and plasma, and finally eluted in 40 μl of elution buffer (Ragusa et al., 2014). RNAs were quantified by fluorometer and spectrophotometer. To profile the transcriptome of 754 miRNAs on TaqMan Low Density arrays (TLDA), 30 ng of serum RNAs were retrotranscribed and pre-amplified, according to the manufacturer's instructions. Pre-amplified products were loaded onto TLDAs, TaqMan Human MicroRNA Array v3.0 A and B (Applied Biosystems|Life Technologies™ Monza, Italy). PCRs on TLDAs were performed on a 7900HT Fast Real Time PCR System (Applied Biosystem|Life Technologies™ Monza, Italy). Results were validated by single TaqMan assays (Applied Biosystems|Life Technologies™ Monza, Italy) using the same amount of RNAs, according to the manufacturer's instructions.

### RNA isolation and RT-PCR from formalin-fixed, paraffin-embedded (FFPE) samples

For miRNAs extraction, eight sections of 20 μm each were cut from FFPE eyes on a RM2245 microtome (Leica, Bannockburn, IL, USA). Sections were transferred to glass slides, and tumor tissue was isolated by hand with a scalpel. We also analyzed FFPE from five unaffected eyes. RNAs were extracted by using Recover All Total Nucleic Acid Isolation Kit (Ambion), following the manufacturer's protocol. The expression of miRNAs from FFPE samples was analyzed by TaqMan MicroRNA Assay, as previously specified.

### Expression analysis

To obtain an accurate miRNAs profiling, we used the global median normalization method (Vallelunga et al., 2014). Based on this method, we identified most stable miRNA (miR-320) to be used as reference gene for data normalization of serum miRNAs in TLDA and single TaqMan assays. For single TaqMan experiments on FFPE samples snRNA U6 was used as reference genes, as already reported (Ragusa et al., 2013).

### Statistical analysis

The unpaired *T*-test was applied to statistically evaluate the expression differences between patients and healthy controls by single TaqMan validation assays. A linear correlation analysis was performed between expression fold changes (FC) of differentially expressed miRNA in FFPE and serum and tumor diameters. Statistical significance was established at a *p* < 0.05.

## Results

### Clinicopathological characteristics of uveal melanomas

This study included a total of 14 patients, 10 women and 4 men, with uveal melanoma. At the time of diagnosis, median age was 61 years (range 29–83 years). The clinicopathological parameters are listed in Table 1. Of the tumors, 8 (57.1%) were located in the choroid and 6 (42.9%) tumors involved the choroid and ciliary body. Histologically, 8 (57.1%) tumors were classified as mixed; 6 (42.9%) as spindle cells. Pathological T stage was pT3b for 1 (7.1%) tumor, 6 (42.9%) was pT4a, 7 (50%) were pT4b. No liver involvement was detected in all cases. The median follow-up period was 15 months. During the follow-up period, no patients died due to the disease progression.

### Profiling of miRNAs from serum in UM patients

**Figure 1:**
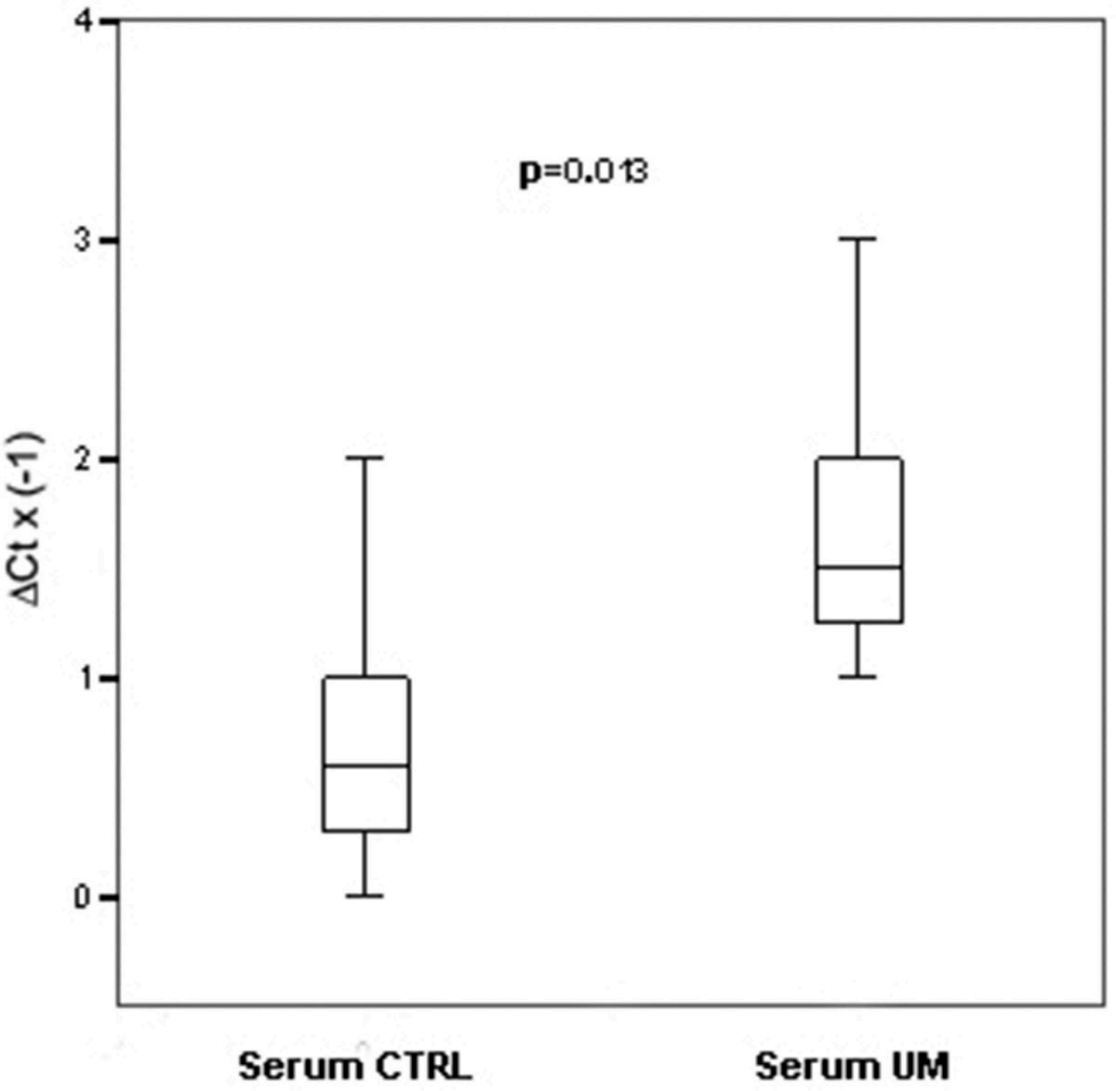
**Single TaqMan assays for miRNA-146a**. Box plots representing the expression of miRNA-146a, analyzed by single TaqMan assay on serum from an independent cohort of 14 patients and 14 controls. y-axis represents the −ΔCt of miRNAs in melanoma patients respect to normal controls. Statistical significance was evaluated by the Wilcoxon rank sum test (*p* = 0.013).

Using TLDA technology, we determined the profiles of 754 miRNAs in serum from 14 patients affected by UM and 14 unaffected controls. Based on SAM analysis (The false discovery rate < 0.05), we found 8 miRNAs differentially expressed compared to normal controls: 2 upregulated miRNAs (miRNA-146a, miRNA-523); 6 downregulated miRNAs (miRNA-19a, miRNA-30d, miRNA-127, miR-451, miR-518f, miR-1274B). We tested by single TaqMan assays specifically the upregulation of miRNA-146a and miR-523 because the most interesting as potential markers of UM. We found that only miRNA-146a was overexpressed in significantly statistical way in serum of UM patients respect to normal controls, (*p* = 0.013; Figure 1). The linear regression analysis did not show a significant correlation between tumor size and FC of miRNA-146a (*r* = 0.146; *P* = 0.688).

### miRNAs expression in FFPE uveal melanoma specimens

**Figure 2:**
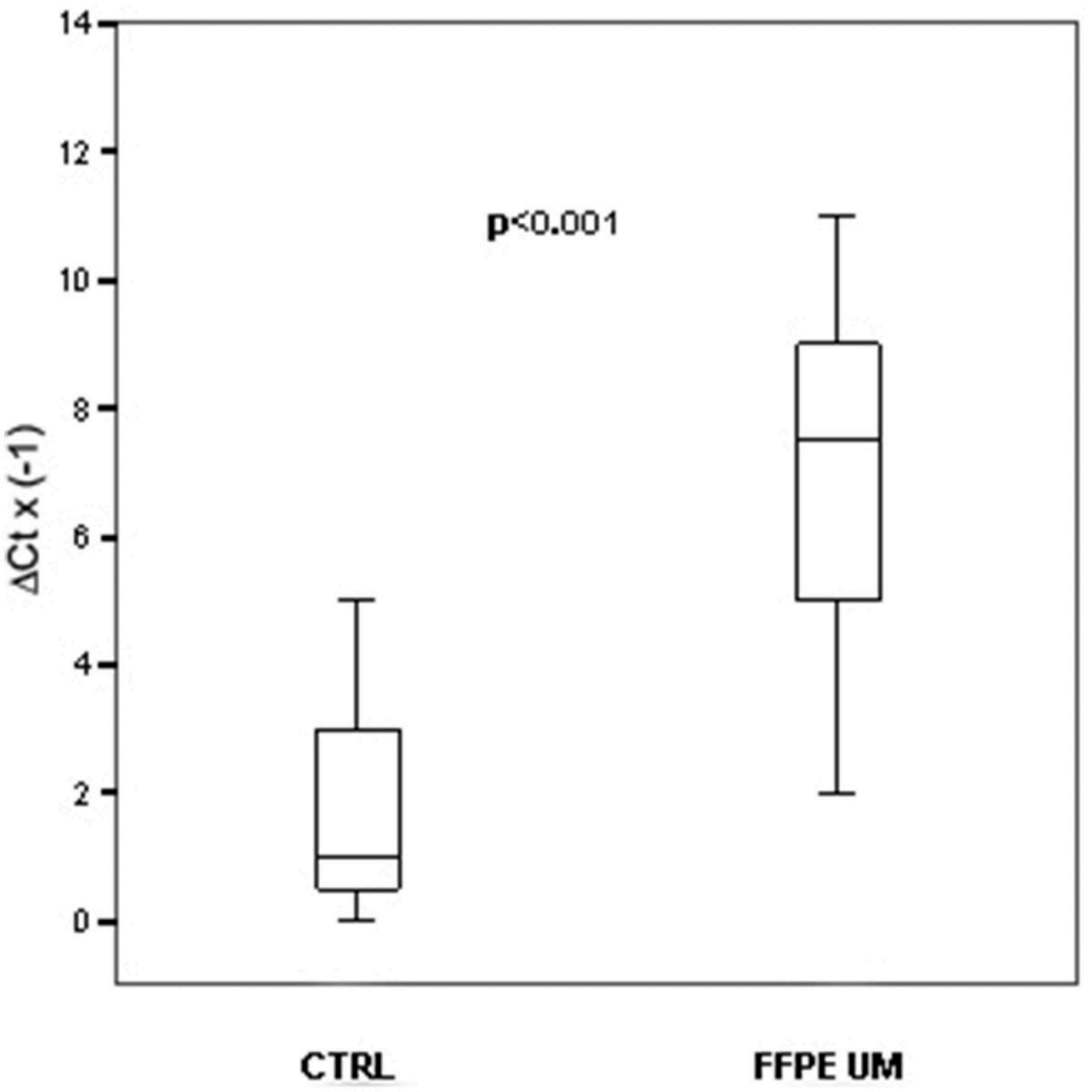
**MiRNAs expression. In FFPE UM specimens**. Box plots representing the expression of miRNA-146a analyzed by single TaqMan assay on paraffin-embedded UM compared to healthy choroidal melanocytes. y-axis represents the −ΔCt of miRNAs in melanoma patients respect to normal controls. Statistical significance was evaluated by the Wilcoxon rank sum test (*p* < 0.001).

To verify whether miRNAs alterations observed in serum of patients with uveal melanoma mirrored some disregulations originating from tumoral cells, we performed single TaqMan assays for miRNA-146a on fourteen formalin-fixed, paraffin-embedded UM samples, and compared them to choroidal melanocytes from five unaffected eyes. Real Time PCR analysis showed that miRNA-146a was statistically upregulated in uveal melanoma cells, as already shown for serum (*p* < 0.001; Figure 2). The linear regression analysis did not show a significant correlation between tumor size and FC of miRNA-146a (*r* = 0.161; *P* = 0.583).

## Discussion

MiRNAs enter into the body fluids with a passive release or an active secretion. Broken and apoptotic cells can release miRNAs through passive route (Turchinovich et al., 2012). However, recent data in the literature reported that cells are also able to secrete miRNAs with microvescicle-dependent or microvescicle-free protein dependent pathways. Two types of microvescicle are secreted from cells: exosomes and shedding vescicle (Simons and Raposo, 2009). Exosomal microvesicles are secreted by a wide variety of cell types and can be detected in different body fluids including the plasma, bronchoalveolar lavage fluid, blood, urine, bile, ascites, breast milk, and cerebrospinal fluid. The higher level of stability in many conditions (e.g., repetitive freeze–thaw cycles, extreme pH, and long-term storage at room temperature) together with development of sensitive non-invasive detection methods make miRNAs a potential disease biomarker. However, some problems remain to be solved. A trustable, approved, and unique internal to normalize the expression pattern of circulating miRNAs between case/control patients must be discovered. Low RNA yield and samples variations can be a problem in next generation sequencing and microarray profiling.

Considering that cancer cells can transfer miRNAs to the extracellular environment, several studies have compared the expression profiles of miRNAs in serum or plasma across a variety of tumors to identify cancer-specific expression patterns. Recent studies have shown that miRNAs can be measured in formalin fixed paraffin embedded (FFPE) tissues (Hoshida et al., 2008). Given the invasive nature of fresh/frozen tissue collection and the availability of FFPE, this serves as a major advance in the feasibility of measuring miRNA levels for the purposes of diagnosis. MiRNA expression profiles have been used to distinguish tumor from normal samples, identification of tissue of origin for tumors of unknown origin or in poorly differentiated tumors and to distinguish different subtypes of tumors. An ideal cancer biomarker must have a specific expression profile that distinguish cancer patients from the normal individuals and must be detect in an early stage of the disease, before clinical symptoms appear. Data obtained suggested that circulating miRNAs expression could be used to discriminate disease samples, demonstrating their potential use as blood-based diagnostic markers of cancers (Fortunato et al., 2014; Ganepola et al., 2014; Mar-Aguilar et al., 2014; Wang et al., 2014). Currently, there are many gaps in our understanding of the function of cell-secreted miRNAs, and many biological events these miRNAs are involved in remain to be elucidated. Two hypotheses are reported in the literature to explain the role of miRNAs in cancer development. The first hypothesis highlights the role of miRNAs in regulating the expression of multiple target mRNAs and therefore control the state of cellular phenotype. In this way, cancer cells could benefit from their own specific nich (Hanahan and Weinberg, 2011). The second hypothesis brings into focus the function of cf. miRNAs in distant organs. Because of incorporation into microparticles and exosomes, miRNAs are very stable and can be measured in the circulation (Ferracin et al., 2010). To develop sensible and specific blood biomarkers of uveal melanoma phenotype, we examined levels of circulating miRNAs in patients with uveal melanoma. By expression profiling we observed a disregulation of 8 serum miRNAs in UM patients, but after single assay validations we found as statistically significant only the overexpression of miRNA-146a. To verify whether miRNAs alterations observed in serum from melanoma patients were the result of molecular dysregulations arising from tumoral cells, we analyzed miRNA-146a on FFPE UM samples and compared them to choroidal melanocytes from five unaffected eyes. MiRNA-146a was statistically upregulated in uveal melanoma cells, as already shown for serum. Therefore, miRNA-146a was the only miRNA upregulated both in the serum and FFPE of melanoma patients suggesting its potential role in neoplastic melanocytes. Changes in miRNA-146a are observed in human diseases such as inflammatory disease (Perry et al., 2008) and cancer (Williams et al., 2008). MiRNA-146a is considered an immune miRNA with potential immune-suppressive role through its recognized target genes, IRAK1 and TRAF6. It is a mediator of inflammation and it is involved in NK cell development and function; its overexpression in NK cells inhibits proliferation and induces apoptosis (Sheedy and O'Neill, 2008; Boldin et al., 2011; Paik et al., 2011). Expression of miRNA-146a is induced by microbial lipopolysaccharide (LPS), IL-1β and TNF-α with an NF-kappaB dependent pathway (Taganov et al., 2006). MiRNA-146a has a role in the complex molecular mechanisms involved in the control of cell growth, differentiation, and survival, processes mainly related to cancer development and progression. MiRNA-146a is overexpressed in papillary thyroid carcinoma (Jazdzewski et al., 2008), pediatric acute lymphoblastic leukemia and pediatric acute myeloid leukemia (Zhang et al., 2009), hepatocellular carcinoma (Xu et al., 2008), and prostate cancer (Xu et al., 2010). miRNA-146a can suppress the metastatic ability of breast cancer cells partially through decreasing constitutive NFκB activity (Bhaumik et al., 2008). Expression of miRNA-146a increases the ability of human melanoma cells to proliferate in culture and form tumors in mice (Forloni et al., 2014). Moreover, miRNA-146a is a target of MITF (Microphthalmia-associated Transcription Factor), a protooncogenic transcription factor acting as a master regulator of melanocyte development, function and survival; it also may be implicated in choroidal melanoma pigmentation and proliferation (Ozsolak et al., 2008; Chen et al., 2011; Yajima et al., 2011). Based on these data, miRNA-146a could have an important regulatory role in the survival of melanocytes from UM.

Main limit of our study was the relatively small number of patients included.

Careful examination by experienced clinicians and diagnostic testing, including angiography, ultrasonography, and magnetic resonance imaging, can be used to establish and confirm the diagnosis. Unfortunately often, distinguishing a small UM from a nevus can be very difficult (Field and Harbour, 2014; Rashid and Grossniklaus, 2014). Effective molecular diagnostic markers for this tumor have not been described, yet. In this paper we demonstrated overexpression of miRNA-146a both in serum and in FFPE; if tumoral cells are able to secrete miRNA-146a, this could be detected in VH, as shown in our previous paper. Unfortunately, detection of circulating miRNAs in VH for diagnostic purposes is not currently feasible because vitreous sampling is an unpleasant and invasive method. However, data shown in this paper suggest the possibility to screen the blood of uveal melanoma patients to find diagnostic miRNAs released by the affected eye. Increased miRNA-146a levels could indicate the neoplastic nature of a lesion, even though further studies will be needed to statistically validate its diagnostic power on larger cohorts of patients.

## Author contributions

MR, AR, RC, LF, VB, AL, LP, TA: conceived the study, wrote the paper, revised intellectual content, and approved final version of the manuscript; MR, AL, AR performed the statistical analysis and interpretation of data; RC and LP performed histological examination.

### Conflict of interest statement

The authors declare that the research was conducted in the absence of any commercial or financial relationships that could be construed as a potential conflict of interest. The handling Editor declared a shared affiliation, though no other collaboration with the authors and states that the process nevertheless met the standards of a fair and objective review.
